# The Influence of Light and Nutrient Starvation on Morphology, Biomass and Lipid Content in Seven Strains of Green Microalgae as a Source of Biodiesel

**DOI:** 10.3390/microorganisms8081254

**Published:** 2020-08-18

**Authors:** Lorenza Rugnini, Catia Rossi, Simonetta Antonaroli, Arnold Rakaj, Laura Bruno

**Affiliations:** 1 LBA-Laboratory of Biology of Algae, Department of Biology, University of Rome “Tor Vergata”, via Cracovia 1, 00133 Rome, Italy; rgnlnz01@uniroma2.it (L.R.); catia.red85@gmail.com (C.R.); 2 Department of Chemical Sciences and Technologies, University of Rome “Tor Vergata”, via della Ricerca Scientifica 1, 00133 Rome, Italy; simonetta.antonaroli@uniroma2.it; 3 Laboratory of Experimental Ecology and Aquaculture, Department of Biology, University of Rome “Tor Vergata”, via Cracovia 1, 00133 Rome, Italy; arnoldrakaj@gmail.com

**Keywords:** *Scenedesmus*, *Tetradesmus*, *Desmodesmus*, FAME, biodiesel, CLSM, Nile red, microalgae

## Abstract

The development of clean and renewable energy sources is currently one of the most important challenges facing the world. Although research interests in algae-based energy have been increasing in the last decade, only a small percentage of the bewildering diversity exhibited by microalgae has been investigated for biodiesel production. In this work, seven strains of green microalgae belonging to the genera *Scenedesmus, Tetradesmus* and *Desmodesmus* were grown in liquid medium with or without a nitrogen (N) source—at two different irradiances (120 ± 20 and 200 ± 20 μmol photons m^−2^ s^−1^)—to evaluate biomass production and FAME (fatty acid methyl esters) content for biodiesel production. The strains of *Tetradesmus obliquus* and *Desmodesmus abundans* grown in N-deprived medium showed the highest FAME content (22.0% and 34.6%, respectively); lipid profile characterization highlighted the abundance of saturated FAME (as C16:0 and C18:0) that favors better viscosity (flow properties) and applicability of biodiesel at low temperatures. Light microscopy and confocal laser scanning microscopy observations were employed as a fast method to monitor the vital status of cells and lipid droplet accumulation after Nile red staining in different culture conditions.

## 1. Introduction

The energy requirements of the transport sector are growing worldwide. Hence, there is a continuous increase in the demand for fuel. In addition, the pollution problems and the impact on the environment of the use of fossil fuels have highlighted the need for alternative energy sources. Development of sustainable, clean and renewable energy sources is, therefore, currently one of the most daunting challenges facing the world. Today, 90% of global energy demand is fulfilled by fossil fuels, which are on the verge of depletion and can be replaced by viable alternatives, such as biofuels [1,2] that could be capable of reducing the petroleum requirement [3]. Photosynthetic microalgae are able to accumulate up to 60–70% of lipids, mostly as triacylglycerols (TAGs) that can be converted into biodiesel via a simple transesterification process [4,5]. Algae biodiesel contains no sulfur and performs as well as petroleum diesel, while reducing emissions of particulate matter, CO, hydrocarbons and SOx [6]. Fatty acid composition is an important factor to consider in the successful generation of biodiesel from algae. The selection of suitable strains of green microalgae that combine high growth rate, good biomass productivity, high lipid content and resistance to stress conditions still remains a challenge for large-scale cultivation and biodiesel production.

Various studies of microalgae have demonstrated that lipid productivity could be altered by several different factors, such as carbon dioxide levels, temperature, light intensity, nutrient concentrations, metal/ion stress [7,8,9]. Light intensity is an important parameter as it has been shown to drastically affect the growth of microalgae and their lipid content: low and high light intensity have been shown to cause unfavorable growth in different species of microalgae, low irradiance induces the formation of polyunsaturated fatty acids (PUFAs) whereas high intensity decreases total polar lipid content [4,10,11,12]. Several studies have demonstrated that nitrogen (N) limitation, even if it slows down the cell growth and development, is the most effective strategy for the production and accumulation of lipids; when N is limited, the proportion of saturated fatty acids (SFAs) and monounsaturated fatty acids (MUFAs) increase and the proportion of polyunsaturated fatty acids (PUFAs) decreases with respect to total lipids [12,13,14]. To date, routine protocols for lipid accumulation in microalgae recommend a two-phase culturing system: first, microalgae are grown in optimized nutritional conditions for maximum biomass production, after which cultures are subjected to nutrient starvation (especially nitrogen), light irradiance variation or the presence of heavy metals to achieve high lipid content in cells.

The *Scenedesmus* and *Desmodesmus* genera are considered ideal microalgal species for biodiesel production due to their high lipid yield and good adaptability—allowing them to grow in different conditions [15]. This study therefore aims to study the effect of two different light irradiance and N deprivation on biomass and lipid production of seven different green strains of microalgae. Strains showing the highest lipid yield were studied in detail, evaluating lipid profile and biodiesel properties that are fundamentally to the success of the algae-based biodiesel industry, in addition to its yields [16]. Few studies have focused on the effects of light intensity combined with N limitation on fatty acid composition. Cell morphology and lipid accumulation were also studied through microscopy observations as a fast tool to analyze the vital status of cells and lipid accumulation. The results illustrated here, will be fundamental for the selection and growth optimization of the most suitable strain of microalgae for the successful production of algae-based biodiesel.

## 2. Materials and Methods

### 2.1. Microalgae Strains

A total of seven strains (Chlorophyceae) were employed in this study. *Scenedesmus acuminatus* 38.81, *S. obliquus* 276.3d and *S. rubescens* 5.95 were supplied by the Culture Collection of Algae at the University of Göttingen, Germany (SAG), while a strain of *Tetradesmus obliquus*, previously known as *Scenedesmus* sp. [17] was isolated from the primary filter of a water purification system and stored in the ‘Vergata Rome University Culture collection’ (VRUC) [18]. *Desmodesmus communis* 276-48 and *D. opoliensis* 64.94 were also obtained from SAG collection, while *D. abundans 283/1.8* was from ACUF (Algal Collection University Federico II). The strains were acclimated in liquid bold basal medium (BBM) [19] for one month (25 mL in 50-mL flasks), under a condition of 120 µmol photons m^−2^ s^−1^, 20 ± 2 °C, relative humidity of 50% and at light–dark regime of 12:12 h.

### 2.2. Experimental Setup

In a first run, cultures were transferred into a 1-L Erlenmeyer flask containing 400 mL of fresh BBM medium (2.5% inoculum) and bubbled with sterilized–filtered air. Growth conditions were the same as those reported above, testing two different irradiances: 120 ± 20 µmol photons m^−2^ s^−1^ (L_120_) and 200 ± 20 µmol photons m^−2^ s^−1^ (L_200_). At the stationary phase, all algal cultures were divided into two aliquots of the same volume (second run) and cells were removed from the medium by centrifugation for 25 min at 19,200× *g* and 36,000× *g* for *Scenedesmus, Tetradesmus* and *Desmodesmus* strains, respectively. After this, the two pellets obtained for each strain were transferred into 400 mL of BBM with a nitrogen source (BBM+N) and in 400 mL of BBM without a nitrogen source (BBM−N) and exposed again at L_120_ and L_200_ (BBM+N/L_120_ and BBM−N/L_200_ growing conditions) until the stationary phase of the second run. All experiments were conducted in triplicates.

### 2.3. Analytical Methods

Optical density (OD) and dry weight (DW) were measured to follow microalgal growth in both run*s*. OD was performed at 560 nm (OD_560_) using a spectrophotometer (Varian Cary 50 Bio UV-Visible spectrophotometer). DWs of the biomass were obtained by filtering the samples through preweighed filters (Whatman GF/C) and then drying them at 104 °C for 24 h before reweighing, to calculate biomass production as gDW L^−1^. Biomass productivity (gDW L^−1^ day^−1^) was calculated by dividing the difference between the dry weights (DWs) at the end and at the start of the experiment by its duration in days, for each run and each tested condition (BBM+N/L_120_ and BBM-N/L_200_). At the end of each experiment, cultures were centrifuged and dried to detect biomass DW expressed in grams.

### 2.4. Lipid Extraction and Fatty Acid Profile Characterization

For lipid extraction, cells were collected by centrifugation for 25 min at 19,200× *g* and 36,000× *g* for *Scenedesmus, Tetradesmus* and *Desmodesmus* strains, respectively and dried at 65 °C for 24 h. Then, biomass obtained by the strains of *S. acuminatus, S. obliquus, S. rubescens* and *T. obliquus* was treated for 5 min with 10 mL of HClO_4_ at 0 °C; biomass from *D. abundans, D. communis, D. opoliensis* was treated for 1 h with 10 mL of HClO_4_ at 0 °C. The difference in the time of exposure to HClO_4_ was due to the presence of three sporopollenin wall layers in *Scenedesmus* species and four in *Desmodesmus* species. After acid treatments, samples were centrifuged at 5000× *g* for 5 min and lipids were extracted by incubating the cells with 15 mL of a mixture made of CHCl_3_/MeOH (2/1, *v*/*v*) for 24 and 48 h for *Scenedesmus, Tetradesmus* and *Desmodesmus* strains, respectively. Then, the obtained organic layer was separated by funnel separation phase, rinsed with water and brine (saturated NaCl solution), and the solvents employed were recovered by rotary evaporation.

The transesterification procedure of lipids to fatty acid methyl esters (FAME) was conducted by boiling 5 mL of crude lipids with 1 mL of MeONa in 0.25 M of MeOH/diethyl ether (1:1, *v*/*v*), following Bannon et al. [20]. Then, 5 mL of brine and 3 mL of hexane/diethyl ether (1:1, *v*/*v*) were added and mixed for 10 min at 50 °C. FAME were then obtained by funnel separation from the organic layer and the FAME profile was determined by gas chromatograph (Varian CP3800) equipped with a split injector and fitted with a fused silica capillary column Supelco SLB-5ms. The standard and the samples (1.0 µL) were injected into the column, with a temperature program starting from 100 °C for 2 min, increased by 5 °C/min to 240 °C final (split/column flow ratio, 1:40). The FAME content was estimated as the percentage of esterified lipids per gram of dry biomass.

### 2.5. Evaluation of Biodiesel Properties

Biodiesel qualities and properties are quantified through standard parameters such as SV (saponification value), IV (iodine value), CN (cetane number), CFPP (cold filter plugging point), degree of unsaturation (DU) and oxidation stability. These parameters were calculated using the empirical Equations (1)–(4) previously reported [8]:

(1) $$\mathrm{SV}\;=\;\sum \left(\frac{560*N}{M}\right)$$

(2) $$\mathrm{IV}\;=\;\sum \left(\frac{254*ND}{M}\right)$$

(3) $$\mathrm{CN}\;=\;46.3\;+\;(\frac{5458}{SV})\;-\;(0.225\;*\;\mathrm{IV})$$

CFPP = (3.1417 ∗ *LCSF*) − 16.477 (4)

where N is the percentage of each FA component, M is the FA molecular mass, D is the number of double bonds, and LCSF is the long-chain saturated factor calculated trough Equation (5):

LCSF = (0.1 × C_16_) + (0.5 × C_18_) + (1 × C_20_) + (2 × C_24_) (5)

The DU was calculated based on Equation (6), as the amount of MUFA and PUFA present in the microalgae oil.

DU = MUFA + 2 × (PUFA)(6)

### 2.6. Microscopy

Morphologic investigations were performed using a light microscope (Zeiss Axioskop; Carl Zeiss, Inc., Thornwood, New York, NY, USA) equipped with a device for differential contrast. Images were acquired using a digital camera NIKON COOLPIX 995. For each strain of the selected *Scenedesmus, Tetradesmus* and *Desmodesmus* genera grown at L_120_ and L_200_, with or without N-source, cell morphology was analyzed taking fifty measurements by ImageJ image analysis software [21]. Lipid droplet accumulation was also observed over the time in the strain of *D. abundans* grown under the different culture conditions. After Nile red staining (100 µg mL^−1^) [22], cells were observed by confocal laser scanning microscope (CLSM; Olympus FV1000) using excitation at 488 and 635 nm and two channels for detecting chlorophyll a (650–750-nm emission range) and lipid labeled by Nile red (maximum emission = 572 nm). Three-dimensional images were constructed from series of 2D cross-sectional images (x-y plane) that were captured at 0.5-μm intervals along the *z*-axis using IMARIS 6.2.0 software (Bitplane AG, Zurich, Switzerland). The regions of interest (ROI) were evaluated in order to perform a spectral analysis in all the visible wavelength regions.

### 2.7. Statistical Analysis

All experiments were performed in triplicates. Principal component analysis (PCA) was performed to evaluate the relationships among the observations (strains in all culture conditions) and the variables: biomass yield, biomass production, lipid yield and FAME. PCA was performed on a correlation matrix with PAST software, version 4.0 [23] and other datasets were processed with GraphPad Prism Software, version 8.0 (San Diego, CA, USA).

## 3. Results and Discussion

### 3.1. Growth Evaluation and Biomass Productivity

**Figure 1:**
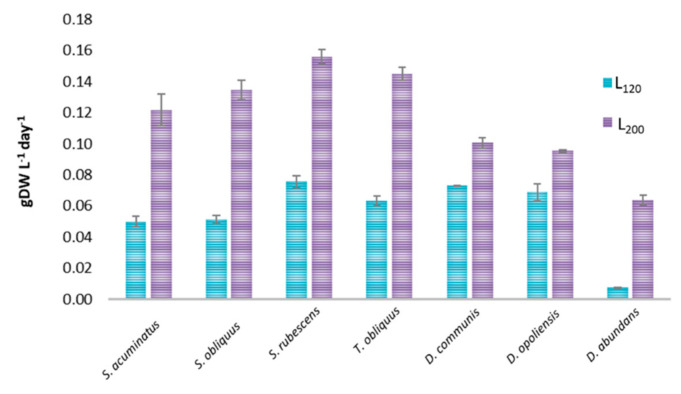
Biomass productivity (gDW L^−1^ day^−1^) obtained during the first run for all the strains employed in this study at the two different irradiances (values are means of 3 measurements ± standard deviation).

Growth curves according to OD_560_ measurements for both runs of all the seven strains are reported in Supplementary Materials (Figures S1 and S2). The cultures showed an exponential phase between 7 and 10 days in complete medium; after the division in two aliquots, the growth in the culture without a nitrogen source was significantly different respect to the ones in the complete medium (*p* < 0.05), although a minimum level of growth was still registered in all the strains tested for around 10–13 days. Biomass productivity of the cultures of *S. acuminatus, S. obliquus, S. rubescens, T. obliquus, D. abundans, D. communis, D. opoliensis* obtained in the first run (Figure 1) and, consecutively, in the second run for all the culture conditions tested was evaluated. For all the strains, the biomass productivity was higher in cultures grown at L_200_ (*p* < 0.05) than L_120_ conditions, in line with data reported by Sforza et al. [24] for *S. obliquus* grown at light irradiance from 10 to 150 µmol photons m^−2^ s^−1^ that obtained the highest growth rate at the maximum light intensity tested. Nzayisenga et al. (2020) [4] demonstrated that the highest biomass production for *Chlorella vulgaris, Ettlia pseudoalveolaris* and *S. obliquus* was at 150 µmol photons m^−2^ s^−1^ and at 300 µmol photons m^−2^ s^−1^ for *Desmodesmus* sp, while the lowest production was at 50 µmol photons m^−2^ s^−1^ for all species, as reported in our study.

**Figure 2:**
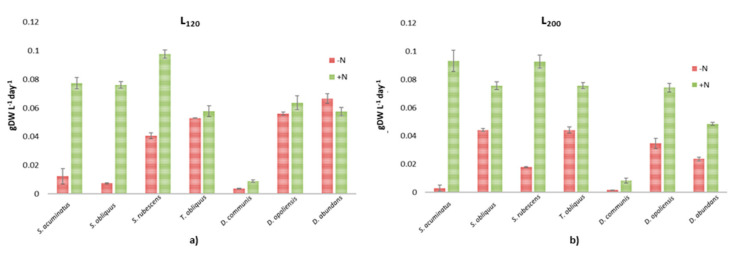
Biomass productivity, as grams of dry weight for liters for day, obtained for the seven strains grown at (**a**) 120 ± 20 µmol photons m^−2^ s^−1^ (L_120_) and (**b**) 200 ± 20 µmol photons m^−2^ s^−1^ (L_200_) with (+N) and without (−N) a nitrogen source.

The effect of nitrogen starvation on the increase in lipid content was evaluated by dividing all cultures in two subcultures, with or without a N-source. Regarding the biomass productivity in this second run (Figure 2), the strain of *S. rubescens* showed the highest values in both culture conditions, particularly at L_200_ (0.930 ± 0.004 gDW L^−1^ in BBM+N/L_120_ and 0.980 ± 0.003 in BBM+N/L_200_, respectively), greater than that obtained among all the strains tested. To evaluate the effect of light and N-source on biomass production and productivity, samples were compared by ANOVA, followed by the Tukey’s multiple comparisons test. Data showed that, in these conditions, there was no significant difference for cultures grown at L_120_ with respect to those grown at L_200_ (*p* > 0.05), but biomass productivity was significantly affected by nitrogen source (*p <* 0.001). Biomass productivity in all *Scenedesmus* and *Tetradesmus* strains was from two to 10 times higher in the complete BBM medium (BBM+N) than in BBM-N in L_120_ and up to 32 times higher when grown in L_200_ conditions for *S. acuminatus*. The presence of a N-source in BBM, also affected the growth of *D. abundans,* that was 5 times greater in BBM+N/L_200_ than BBM-N/L_200_.

Thus, all the *Scenedesmus, Tetradesmus* and *Desmodesmus* strains tested in this study showed that the best growth conditions among the ones tested, can be represented by complete BBM and an exposure to 200 ± 20 µmol photons m^−2^ s^−1^.

### 3.2. Lipid Yield and FAME Characterization

Many stress factors have been shown to affect biomass productivity in different species of microalgae and thus lipid accumulation, such as N and P limitation, light irradiance and the presence of heavy metals [17,25,26]. For many species of microalgae, it has been established that N-deprivation correlates positively with lipid accumulation which in turn may result in a lower biomass yield. Thus, it is a good practice to produce biomass in optimal culture conditions for the species under study and then apply the stress factor to increase lipid content. In this study, biomass production and FAME yield obtained for all the strains of *Scenedesmus, Tetradesmus* and *Desmodesmus* under the selected growth conditions, are reported in Table 1. After lipid extraction and transesterification, FAME obtained were quantified and characterized. FAME yields in BBM-N were significantly higher than in BBM+N (*p* < 0.05). In general, *Desmodesmus* strains showed higher production of FAME than *Scenedesmus* and *Tetradesmus* strains, up to 34.60% in *D. abundans* when grown in BBM-N/L_200_, while *T. obliquus* sp. and *S. rubescens* had the highest FAME yields when grown in BBM-N/L_120_.

Biodiesel quality is related to fatty acid (FA) composition and is determined by the degree of saturation of the fatty acid. In this study, FAME characterization was conducted on the microalgal strains that showed the highest FAME yields, *T. obliquus* and *D. abundans*, being the most interesting, at all the culture conditions tested (Table 2). GC profiles showed that saturated fatty acids (SFAs) were over 90% of total FAME, in *T. obliquus* and *D. abundans* grown in BBM-N/L_120_ and BBM+N/L_200_, respectively. The ideal FAME component for good quality biodiesel should be rich in palmitic (C16:0) and stearic acids (C18:0): a high percentage of saturated FAME favors better viscosity (flow properties) and the applicability of biodiesel at low temperatures [27]. In this study, C16:0 and C18:0 were detected in all culture conditions in both strains, with a percentage of up to 81% for C16:0 and 15% for C18:0. These fatty acids are present in all cyanobacteria and in many other microalgae studied to date [28,29,30], in addition to being recognized as the most interesting for the production of biodiesel. Between monounsaturated FAME (MUFA), gadoleic acid (C20:1) was the most abundant for *T. obliquus* grown in BBM+N/L_120_ (about 21%) and for *D. abundans* in BBM-N/L_120_ (over 40%). Long chain saturated and MUFA are suitable for biodiesel, as they improve oxidative stability without greatly affecting its cold flow properties [8,31], while the reactive sites of polyunsaturated fatty acids (PUFA) are susceptible for free radical attack and, in high quantities, negatively affect the oxidative stability of biodiesel [32]. Linolelaidic and linoleic acid (C18:2 and C18:3), were both found in a percentage higher than 10% in *D. abundans* in BBM+N/L_120_, while PUFA with more than 4 double bonds never exceed 1% (according to the European standard EN 14,214 [33]), except for *D. abundans* in BBM-N/L_200_.

Comparing the biodiesel properties obtained here with the European biodiesel (EN 14214) and American biodiesel standards (ASTM D 6751-08) [34], CN of cultures grown in absence of N, both at L_120_ and L_200_, were in accordance with the requirements of US standards, that requires CN > 37 and IV < 120 (detected in all culture conditions for both species) for good biodiesel quality. A high CN value is an indicator of better combustion and low nitrous oxide emissions, the IV is used to determine the degree of unsaturation of biodiesel oil. The ratio between unsaturated fatty acids (UFAs) and saturated fatty acids (SFAs) is crucial for the evaluation of biodiesel physical properties. Here, the smallest ratio UFAs/SFAs between the tested conditions was measured in *Scenedesmus* sp. in BBM-N/L_120_ and *D. abundans* in BBM-N/L_200_ (UFAs/SFAs = 0.07), indicating a high percentage of SFAs that are responsible for resistance to degradation and oxidation, improving shelf life [35,36]. Degree of unsaturation (DU) influences the oxidative stability of biodiesel and it is the sum of the masses of MUFA and PUFA. EN 14,214 and ASTM D6751-08 have not mentioned the value of DU, but it influences the IV of biodiesel that should be <120 [32]. Except for *D. abundans* in BBM+N/L_120_, the IV values calculated in this study are in accordance with the European and American standards for both species in all the culture conditions.

### 3.3. Microscopy Observations

When culturing microalgae, for biodiesel production, the availability of a quick method to monitor the status of the culture could be a useful and cost-saving tool. Thus, in the present study we monitored the cell morphology and the lipid accumulation by using light and confocal laser scanning microscopes. Light microscopy (LM) observations were carried out for all the strains investigated at the start of the experiment and during the stationary phase of growth (between 10th and 13th day) to evaluate any significant variations in cell size and morphology depending on the different culture conditions (Table 3).

The deprivation of a nitrogen source induced a variation of the cell size at the stationary phase, with the highest increase in cell length size in *S. acuminatus* (from 20.55 ± 2.51 µm at the initial phase to 24.49 ± 2.90 at stationary phase), *D. abundans* (from 6.71 ± 1.54 µm to 8.13 ± 1.27 µm) and *D. opoliensis* (from 21.55 ± 1.88 µm to 29.07 ± 1.69 µm). Various studies have shown that the genera *Scenedesmus, Tetradesmus* and *Desmodesmus* are pleomorphic with high morphologic plasticity. All genera are able to produce unicells and/or coenobia under various environmental conditions (e.g., length of photoperiod, pH, nutrients, etc.) and in response to predators [37,38,39]. Pancha et al. [13] observed a change in cell size of *Scenedesmus* sp. CCNM 1077 when grown in nitrate starved conditions, from 4.5 µm in complete medium to around 5.3 µm after 15 days of N-deprivation, around 16% greater than control, as for *S. acuminatus* and *D. abundans* in this study. Cell sizes of *D. opoliensis* grown in BBM-N showed a 25% increase compared with BBM+N, like the results reported for N-deprived *Nannochloropsis* sp. and *Chlorococcum* sp. cells by Yap et al. [40] that were approx. 25% bigger than the N-replete cells.

**Figure 3:**
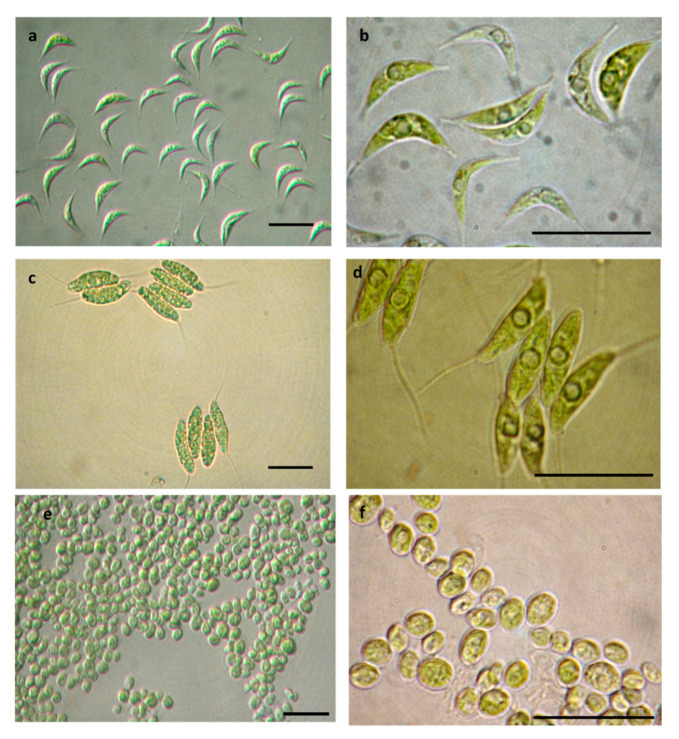
Light microscopy (LM) observations of *S. acuminatus* SAG 38.81 grown in BBM+N/L_200_ (**a**) at the start and (**b**) at stationary phase; *D. abundans* ACUF 283/1.8 in BBM+N/L_200_ (**c**) at the start and (**d**) at stationary phase; *D. opoliensis* SAG 64.94 in BBM-N/L_120_ (**e**) at the start and (**f**) at stationary phase. bar = 10 µm.

All the strains investigated appeared green in color (Figure 3a,c,e) in presence of the nitrogen source (BBM+N) while the nitrogen starvation induced a shift to a yellow green color of the cells in all the cultures grown in BBM-N (Figure 3b,d,f). The change in color was a clear effect of the stress conditions induced by N-limitation. This triggers many metabolic responses in microalgae, such as the degradation of nitrogenous compounds like proteins, chlorophyll, DNA, etc. and accumulation of energy rich compounds like lipids and carbohydrates [10,14].

**Figure 4:**
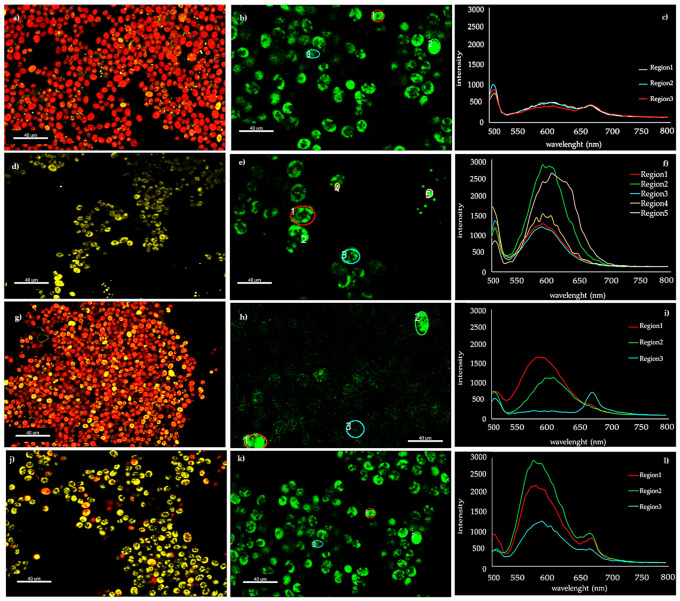
Confocal laser scanning microscope (CLSM) observations of *Desmodesmus abundans.* (**a**–**c**) cells grown in BBM+N/L_120_; (**d**–**f**) cells grown in BBM-N/L_120_; (**g**–**i**) cells grown in BBM+N/L_200_; (**j**–**l**) cells grown in BBM-N/L_200_. Colored circles in (**b**,**e**,**h**,**k**) represent the regions of interest (ROI) of interest (from 1 to 5) studied by spectral analyses in the scan visible region (**c**,**f**,**i**,**l**).

To evaluate lipid accumulation, the use of staining dyes (e.g., Nile red, BODIPY) offer a rapid analysis tool for a qualitative estimation of neutral lipid content, avoiding time-consuming and costly gravimetric analyses [41,42]. Confocal Laser Scanning Microscope (CLSM) observations of lipid droplets in microalgal cells after Nile red staining were performed on the culture of *D. abundans* that showed the highest yield of FAME. In cultures grown in BBM+N both at L_120_ and L_200_, cells appeared red in color due to the autofluorescence of chlorophyll and just a few lipid droplets, identified by their yellow color, were visible (Figure 4a,g, respectively). By contrast, when *D. abundans* was grown in BBM-N, cultures showed evident yellowish spots inside cells due to the presence of neutral lipids, both in L_120_ and L_200_ conditions (Figure 4d,j, respectively). With the confocal microscopy, the analysis of the in vivo fluorescence spectra in small detection areas can be investigated, so that single cells can be studied in vivo using a noninvasive technique for obtaining qualitative information [43]. In this study, the spectral analysis was performed in selected Regions Of Interest (ROI) representing single cells or subcellular regions (Figure 4b,e,h,k) to evaluate the maximum emission intensity of Nile red (between 580 and 600 nm) in correspondence of lipid granules. As indicated in Figure 4e,f,k,l (ROI = 4 and ROI = 1, respectively) in N-depleted lipid-rich cells the emission peaks corresponded to Nile red fluorescence, while in cells grown in complete medium that resulted without evident lipid droplets (Figure 4b,c,h,i, ROI = 3) the emission peaks corresponded to pigment autofluorescence (505 to 670 nm) of chlorophyll *a*. These results are in line with those reported by Gusbhet et al. [44] that observed a specific emission peak at 598 nm—when excited at 495 nm—in Nile red stained cells of *Auxenochlorella protothecoides,* while both stained and unstained cells also have an emission peak of 678 nm, due to the autofluorescence of chlorophyll *a.* Tracking the increase in lipid content during the growth of microalgae represents a useful tool that could contribute to the cost reduction needed to reach the goal of the sustainable and economical production of biodiesel [45]. Different authors [41,46] described some quantitative protocols that exploit the possibility to correlate total lipids content with cell fluorescence, using fluorimetry and/or spectrometry methodologies.

### 3.4. Multivariate Analysis

**Figure 5:**
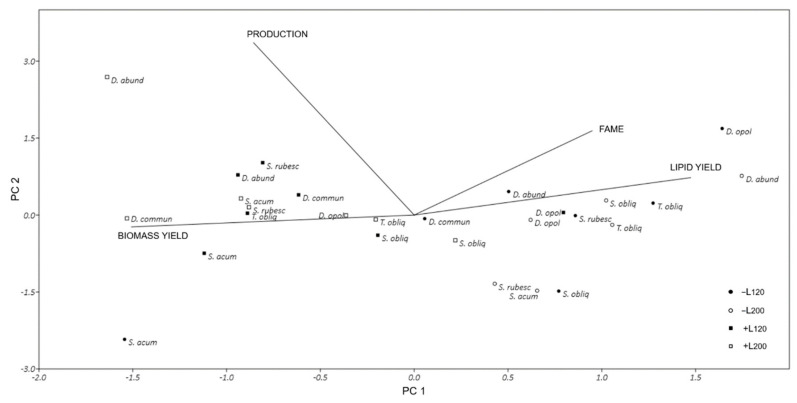
Principal component analysis (PCA) performed on a correlation matrix evidencing the relationships among observations (all the strains in all culture conditions) and variables. Biomass means dry weight as grams; production means biomass production as gDW L^−1^; lipid yield and FAME expressed as %. −/+ indicated the absence/presence of N in BBM medium.

A principal component analysis (PCA) was performed on a dataset of 27 observations (all the strains in all culture conditions) and 4 variables (dry weight of the biomass, biomass production, lipid yield and FAME) (Figure 5). The PCA allowed to ordinate the observations in a biplot evidencing the treatment groups with existing correlation among the original variables. The first Axis (PC1) explained 52.5% and the second axis (PC2) 21.4% of the variance. Therefore, the two-axis ordination biplot described 73.9% of the total variance. Harvested biomass was negatively correlated with FAME and lipid yield and all provided the heaviest loading on axis 1 while the biomass production was negatively correlated with lipid yield having the heaviest loading on axis 2. Axis 1 separated PCAs and clearly discriminated between the BBM+N and BBM-N. All observations for cultures grown in BBM-N were located on the right-hand side of the graph, indicating high FAME and lipid yields and low biomass production in these culture conditions. Instead, observations related to cultures grown in BBM+N, were located on the left of the graph, characterized by high biomass production with a low FAME and lipid yields. The PCAs, however, failed to clearly discriminate between L_120_ and L_200_ conditions confirming that the two light irradiances used in this work were unable to induce significant differences in the growth and lipid production of these strains.

## 4. Conclusions

In this study, seven strains of green microalgae were evaluated in terms of biomass productivity, FAME yields and biodiesel properties. *T. obliquus* and all the species of *Desmodesmus* genus, showed the highest biomass production in the tested growth conditions, when grown in BBM+N at 200 ± 20 µmol photons m^−2^ s^−1^ of light intensity. The highest biomass production for *T. obliquus* also corresponded to high FAME yield (up to 23%), while among the *Desmodesmus* genus, *D. opoliensis* and *D. abundans* showed a FAME yield between 25% and 35% in BBM-N/L_200_. FAME profile characterization of *T. obliquus* and *D. abundans* demonstrated that saturated fatty acids (SFAs) were over 90% of total FAME and were rich in C16:0 and C18:0 that favors better viscosity and the applicability of a good quality biodiesel at low temperatures. The accordance of biodiesel properties obtained here with European biodiesel (EN 14,214) and American biodiesel standards (ASTM D 6751-08), also reinforced the suitability of these microalgae for the production of lipids capable of fulfilling all the requirements for a top-quality biodiesel. Moreover, evaluation of cell morphology and size-increase represents a reliable way of monitoring the cell culture conditions when exposed to stressing conditions such as nitrogen starvation. This, coupled with the observation of the increase in fluorescence intensity of lipid droplets in stained cells of *D. abundans,* after some days following the nitrogen starvation, suggested the possibility of evaluating lipid accumulation in vivo over time, in order to better estimate maximum lipid production before the direct extraction of these compounds. Therefore, *T. obliquus* and *D. abundans* seem to be promising candidates for further studies on their applicability as an alternative sustainable and renewable source of biofuels.

## Figures and Tables

**Table 1:** Biomass production (in terms of gDW L^−1^) and fatty acid methyl esters (FAME) yields (as percentage, %) obtained both for *Scenedesmus, Tetradesmus* and *Desmodesmus* strains in different culture conditions (BBM=bold basal medium).

		*S. acuminatus*	*S. obliquus*	*S. rubescens*	*T. obliquus*	*D. abundans*	*D. communis*	*D. opoliensis*
**BBM-N/L_120_**	gDW L^−1^	0.08 ± 0.00	0.05 ± 0.02	0.36 ± 0.01	0.37 ± 0.01	0.52 ± 0.05	0.62 ± 0.03	0.73 ± 0.01
	FAME (%)	4.56	13.01	18.80	22.26	22	8.67	23.00
**BBM+N/L_120_**	gDW L^−1^	0.54 ± 0.01	0.53 ± 0.03	0.88 ± 0.04	0.40 ± 0.02	0.92 ± 0.09	0.70 ± 0.01	0.63 ± 0.03
	FAME (%)	6.60	8.37	16.13	11.52	10.51	16.29	3.53
**BBM-N/L_200_**	gDW L^−1^	0.02 ± 0.01	0.35 ± 0.02	0.14 ± 0.01	0.35 ± 0.01	0.27 ± 0.01	0.42 ± 0.02	0.28 ± 0.01
	FAME (%)	16.88	7.04	11.92	14.79	34.60	n.d.	25.10
**BBM+N/L_200_**	gDW L^−1^	0.75 ± 0.07	0.61 ± 0.01	0.74 ± 0.04	0.61 ± 0.02	1.41 ± 0.07	0.82 ± 0.02	0.58 ± 0.01
	FAME (%)	12.24	14.34	8.34	8.85	17.88	3.93	13.75

n.d.—not determined.

**Table 2:** FAME profile of the two green microalgae that showed the highest lipid yields in different culture conditions.

FAME	BBM-N/L_120_		BBM+N/L_120_		BBM-N/L_200_		BBM+N/L_200_	
	*T. obliquus*	*D. abundans*	*T. obliquus*	*D. abundans*	*T. obliquus*	*D. abundans*	*T. obliquus*	*D. abundans*
C14:0	0.00	0.00	1.58	0.71	0.00	0.47	0.94	0.44
C16:0	81.75	46.18	55.07	31.79	62.94	73.71	50.90	32.72
C16:1 (9)	0.00	0.66	2.85	0.92	0.00	1.39	1.19	1.16
C18:0	11.62	7.01	3.76	2.58	15.54	15.76	6.26	6.73
C18:1 (trans-9)	4.11	2.90	1.50	0.00	0.00	0.62	1.22	0.59
C18:1 (cis-9)	0.00	0.00	0.00	0.00	0.00	0.00	0.00	42.29
C18:2 (trans-9,12)	2.52	0.00	0.00	8.85	0.00	0.00	31.45	0.00
C18:2 (cis-9,12)	0.00	0.00	0.00	13.83	0.00	0.00	6.55	6.36
C18:3 (9,12,15)	0.00	0.54	14.31	39.12	3.72	0.00	0.00	0.00
C20:0	0.00	0.00	0.00	0.00	0.00	2.78	0.00	0.00
C20:1	0.00	42.72	20.94	2.19	17.80	2.23	0.00	9.71
C20:4 (5,8,11,14)	0.00	0.00	0.00	0.00	0.00	3.02	0.00	0.00
**SFA (%)**	93.37	53.19	60.41	35.09	78.48	92.73	58.10	39.89
**MUFA (%)**	4.11	46.27	25.29	3.11	17.80	4.25	2.41	53.75
**PUFA (%)**	2.52	0.54	14.31	61.80	3.72	3.02	38.00	6.36
***SV***	214.20	199.70	207.18	206.22	207.63	211.22	209.07	203.50
***IV***	8.08	39.56	60.42	150.86	24.74	12.46	39.56	59.16
***CN***	44.51	37.43	32.73	12.38	40.76	43.52	30.08	33.02
***CFPP * (°C) **	27.46	9.04	6.73	−2.43	27.71	40.19	9.04	4.37
***LCSF***	13.99	8.12	7.39	4.47	14.06	18.04	8.22	6.64
***DU***	9.15	47.35	53.90	126.71	25.24	10.29	76.41	66.48

**Table 3:** Evaluation of cell length for all strains in the tested conditions, measured at the initial phase of the experiment and at the stationary phase. Values are means of fifty measures ± standard deviations.

	Growth Conditions	Length (µm)	
		*Initial Phase*	*Stationary Phase*
*S. acuminatus*	BBM-N/L_120_	20.55 ± 2.51	24.49 ± 2.90
	BBM+N/L_120_	19.68 ± 4.22	25.45 ± 3.09
	BBM-N/L_200_	26.58 ± 3.71	26.75 ± 2.64
	BBM+N/L_200_	25.72 ± 3.91	26.03 ± 3.92
*S. obliquus*	BBM-N/L_120_	12.69 ± 1.95	13.41 ± 3.18
	BBM+N/L_120_	15.05 ± 2.21	13.46 ± 2.58
	BBM-N/L_200_	18.02 ± 2.83	14.73 ± 4.06
	BBM+N/L_200_	16.22 ± 2.61	17.14 ± 4.30
*S. rubescens*	BBM-N/L_120_	12.04 ± 2.18	11.83 ± 2.36
	BBM+N/L_120_	12.07 ± 2.26	11.78 ± 2.30
	BBM-N/L_200_	14.25 ± 3.01	13.58 ± 2.68
	BBM+N/L_200_	13.72 ± 2.35	13.86 ± 1.74
*T. obliquus*	BBM-N/L_120_	13.84 ± 2.10	16.18 ± 2.41
	BBM+N/L_120_	16.23 ± 3.26	15.21 ± 1.28
	BBM-N/L_200_	18.93 ± 2.27	18.28 ± 3.20
	BBM+N L_200_	18.77 ± 3.14	15.27 ± 2.64
*D. abundans*	BBM-N/L_120_	5.85 ± 1.44	5.48 ± 1.08
	BBM+N/L_120_	6.33 ± 1.42	5.20 ± 1.15
	BBM-N/L_200_	6.71 ± 1.54	8.13 ± 1.27
	BBM+N/L_200_	6.32 ± 1.32	6.58 ± 1.16
*D. communis*	BBM-N/L_120_	22.74 ± 2.37	20.16 ± 2.63
	BBM+N/L_120_	22.76 ± 1.68	19.13 ± 2.42
	BBM-N/L_200_	21.81 ± 3.65	21.65 ± 2.96
	BBM+N/L_200_	21.23 ± 2.40	20.87 ± 2.64
*D. opoliensis*	BBM-N/L_120_	29.14 ± 3.08	29.88 ± 1.89
	BBM+N/L_120_	27.65 ± 3.69	24.02 ± 2.55
	BBM-N/L_200_	21.55 ± 1.88	29.07 ± 1.68
	BBM+N/L_200_	24.49 ± 2.42	24.17 ± 2.35

## Supplementary Materials

The following are available online at https://www.mdpi.com/2076-2607/8/8/1254/s1, Figure S1: Growth curves of the strains belonging to the genera *Scenedesmus* and *Tetradesmus*; Figure S2: Growth curves of the strains belonging to the genera *Desmodesmus*.
