# Supplementary material for: The Influence of Light and Nutrient Starvation on Morphology, Biomass and Lipid Content in Seven Strains of Green Microalgae as a Source of Biodiesel
Source: Microorganisms. 2020 Aug 18;8(8):1254. doi: 10.3390/microorganisms8081254 (PMC7464752; doi:10.3390/microorganisms8081254)
Supplement: Supplementary file 1 [file microorganisms-08-01254-s001.pdf]

*Article*

# THE INFLUENCE OF LIGHT AND NUTRIENT STARVATION ON MORPHOLOGY, BIOMASS AND LIPID CONTENT IN SEVEN STRAINS OF GREEN MICROALGAE AS A SOURCE OF BIODIESEL

**Lorenza Rugnini [1], Catia Rossi[1], Simonetta Antonaroli[2], Arnold Rakaj[3] and Laura Bruno[1,*]**

[1] LBA-Laboratory of Biology of Algae, Dept. of Biology, University of Rome "Tor Vergata", via Cracovia 1, 00133, Rome, Italy; rgnlnz01@uniroma2.it, laura.bruno@uniroma2.it, catia.red85@gmail.com

[2] Department of Chemical Sciences and Technologies, University of Rome "Tor Vergata", via della Ricerca Scientifica 1, 00133, Rome, Italy; simonetta.antonaroli@uniroma2.it

[3] Laboratory of Experimental Ecology and Aquaculture, Department of Biology, University of Rome "Tor Vergata", via Cracovia 1, 00133, Rome, Italy, arnoldrakaj@gmail.com

* Correspondence: laura.bruno@uniroma2.it; Tel.: +39 3383784941

**Table of content** **Pages**

**Figure S1-** Growth curves of the strains belonging to the genera *Scenedesmus* and *Tetradesmus* **2**

**Figure S2-** Growth curves of the strains belonging to the genera *Desmodesmus* **3**

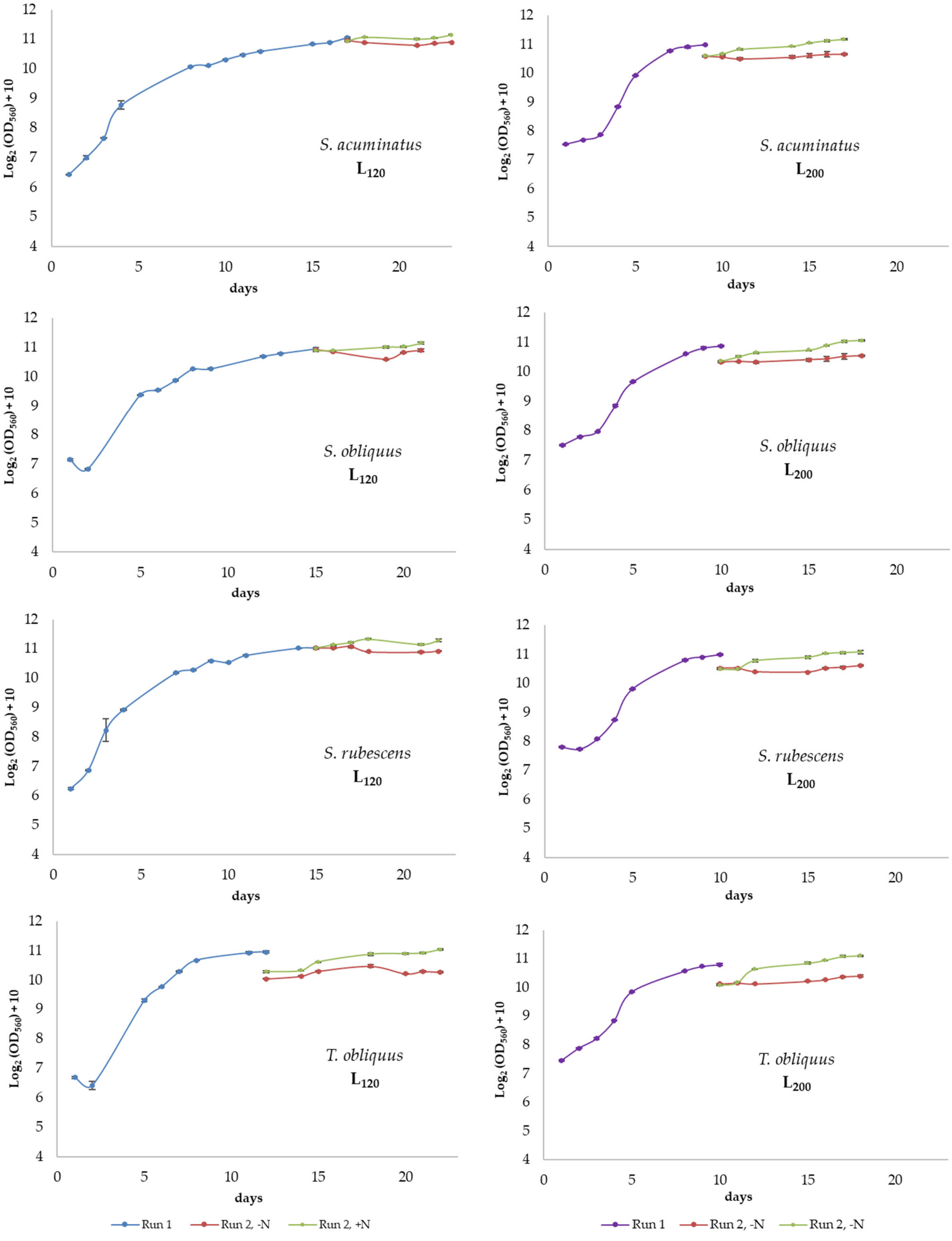


**Figure S1.** Growth curves of *S. acuminatus*, *S. obliquus*, *S. rubescens* and *T. obliquus* at the two different irradiances ($L_{120}$: 120 µmol photons $m^{-2}$ $s^{-1}$; $L_{200}$: 200 µmol photons $m^{-2}$ $s^{-1}$) as logarithm of OD detected at 560 nm versus time (days). Cultures obtained during run 1, were divided in two sub-culture with (+N) or without (-N) nitrogen for each culture conditions tested in run 2.

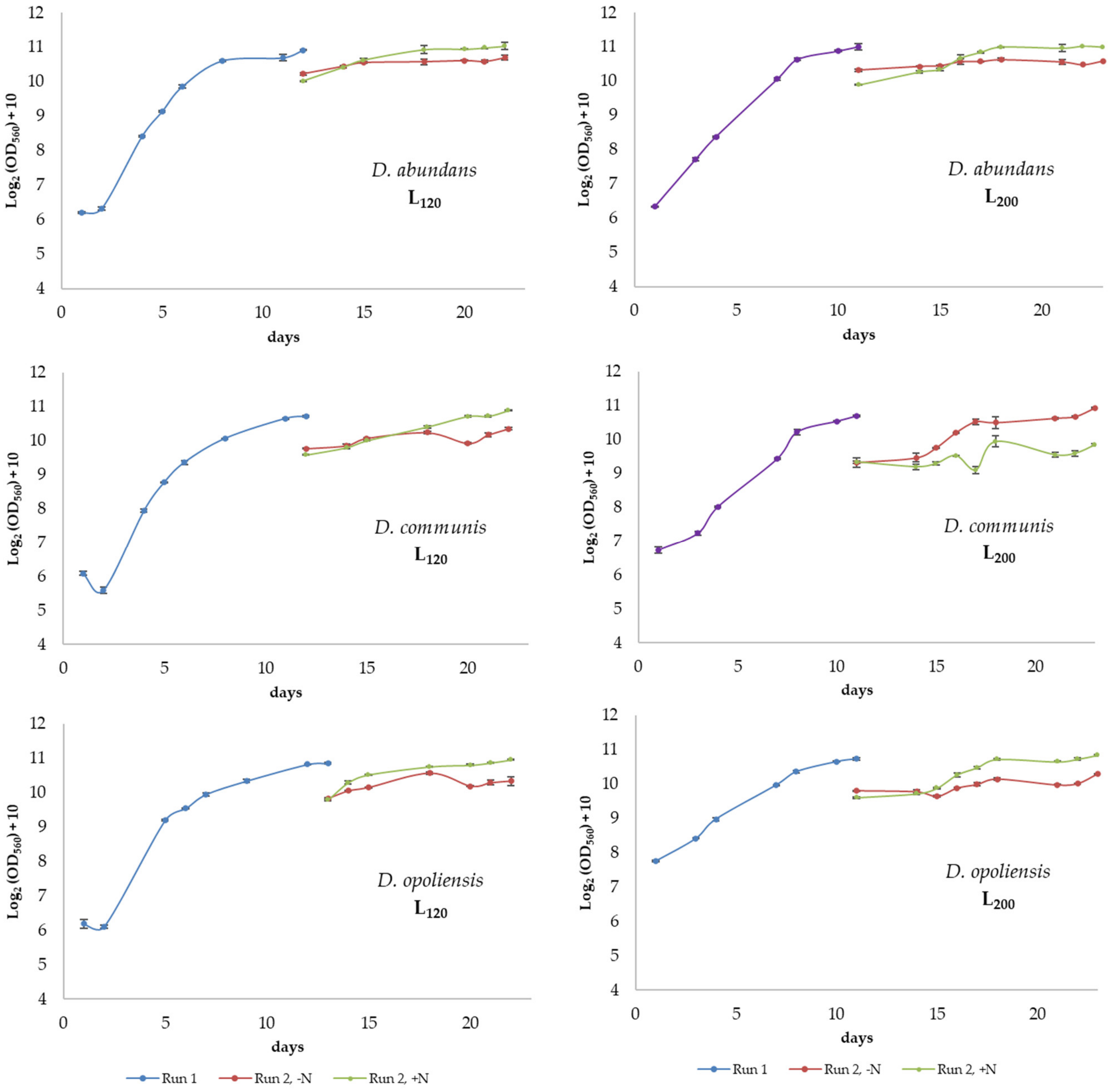


**Figure S2.** Growth curves of *D. abundans*, *D. communis* and *D. opoliensis* grown at the two different irradiance (L120: 120 µmol photons $m^{-2}$ $s^{-1}$; L200: 200 µmol photons $m^{-2}$ $s^{-1}$) as logarithm of OD detected at 560 nm versus time (days). Cultures obtained during run 1, were divided in two sub-culture with (+N) or without (-N) nitrogen for each culture conditions tested in run 2.
